# Commentary: “Association between diabetes mellitus, prediabetes and risk, disease progression of Parkinson's disease: a systematic review and meta-analysis”

**DOI:** 10.3389/fnagi.2023.1223636

**Published:** 2023-06-15

**Authors:** Michael Lawton, Yoav Ben-Shlomo, Dilan Athauda, Naveed Malek, Donald G. Grosset

**Affiliations:** ^1^Department of Population Health Sciences, University of Bristol, Bristol, United Kingdom; ^2^Neurodegeneration Biology Laboratory, Francis Crick Institute, London, United Kingdom; ^3^Department of Clinical and Movement Neurosciences, UCL Queen Square Institute of Neurology, London, United Kingdom; ^4^Department of Neurology, Queen's Hospital, Romford, United Kingdom; ^5^School of Psychology and Neuroscience, University of Glasgow, Glasgow, United Kingdom

**Keywords:** diabetes mellitus, Parkinson's disease, disease progression, cohort studies, meta-analysis

## Introduction

Finding factors that are causally related to both risk of Parkinson's and/or progression of Parkinson's disease (PD) are of considerable interest. This could help in finding new treatments or the repurposing of existing treatments from other diseases as well as assist in counseling of recently diagnosed patients. One factor that has been of interest for many years in the epidemiology of PD is Diabetes.

Systematic reviews and meta-analyses are methods that are used to summarize the evidence across many publications. Zhong and Wang present a systematic review and meta-analysis of the association between diabetes, prediabetes and risk, disease progression of Parkinson's (Zhong and Wang, 2023). This paper is a commentary about problems with some of the meta-analyses presented in that article.

## Tracking Parkinson's cohort

The Tracking Parkinson's cohort is a large, prospective, observational cohort of individuals who were recently diagnosed with Parkinson's (Malek et al., 2015). Recruitment was completed between February 2012 and May 2014 from multiple centers across the United Kingdom. Participants had to be within 3.5 years of diagnosis at recruitment and have clinic visits every 18 months where they go through a large battery of questionnaires completed by either doctors, nurses or themselves. These questionnaires rate the severity of many motor and non-motor features of Parkinson's and include both the Movement Disorder Society Unified Parkinson's Disease Rating Scale (MDS-UPDRS) and the Montreal Cognitive Assessment (MoCA).

We have published two papers that have looked at the effect of Diabetes Mellitus (DM) on patients within the Tracking Parkinson's cohort. The first, published in 2016, was a cross-sectional study of only the baseline data comparing the severity of motor and cognitive disease in those with and without different vascular risk factors one of which was DM (Malek et al., 2016), including both Type 1 and Type 2 DM. This first paper also restricted the analysis of motor and cognitive disease to those without a history of stroke, transient ischaemic attack or cardiac disease. The second, published in 2022, was a longitudinal study that looked at the effect of baseline Type 2 DM on subsequent disease progression (Athauda et al., 2022).

## Problems with use of Tracking Parkinson's cohort data in the meta-analysis

One of the meta-analyses that is presented in Zhong and Wang's article looks at the association between DM and motor progression of PD (figure 3 in the original article) and another looks at the association between DM and cognitive decline (figure 5 in the original article). There are three issues with these meta-analyses.

- Double counting: the results from the Athauda and Malek papers are presented as two individual studies. However since they come from the same cohort they are clearly not independent and the Tracking Parkinson's cohort will be given a greater weighting in the meta-analysis than it should have.
- Different effect estimates: in the meta-analysis of DM and motor progression of PD, they incorrectly combine absolute and relative effect estimates where one is from a cross-sectional analysis and the other from a longitudinal analysis. The result they report from the Malek paper, 3.65 (95% CI: 1.07 to 6.22), is an adjusted mean difference from a linear regression of baseline MDS-UPDRS III total scores. Whilst the result they report from the Athauda paper, 1.55 (95% CI: 1.07 to 2.23) is an adjusted hazard ratio from a Cox regression model where substantial gait impairment was the outcome (defined as score > 3 in MDS-UPDRS III question 10). These statistics are clearly not comparable and are also both incorrectly labeled as Risk Ratios in the forest plot.
- Binary vs. ordinal outcomes: in their meta-analysis of DM and cognitive decline they again combine two different effect estimates where one is from a cross-sectional analysis and the other from a longitudinal analysis. In the Malek paper, the effect estimate of 1.52 (95% CI: 0.89 to 2.58) is an adjusted odds ratio from a ordinal logistic regression where Normal Cognition, Mild Cognitive Impairment (MCI) and Dementia (0, 1, 2) at baseline was the outcome (defined using the MoCA and MDS-UPDRS I question 1). Hence this is the impact of DM in moving up one category on the ordinal scale. Whilst the result they report from the Athauda paper, 1.74 (95% CI: 1.19 to 2.55), is an adjusted hazard ratio from a Cox regression model for a binary outcome of MCI (defined using the MoCA and MDS-UPDRS I question 1). These statistics are clearly not comparable and are also both incorrectly labeled as Risk Ratios in the forest plot. Also, it should be noted that slightly different cut-points were used in these two papers to define MCI using the MoCA and we have planned research that is going to consider the optimal cut-point comparing sensitivity and specificity.

## Discussion

Given that the authors have made mistakes in reporting from our two papers it is possible that there have been some mis-representations of other papers that have been cited. We would recommend that they carefully consider the other papers that have been included in the meta-analyses and submit an erratum with the revised results.

## Author contributions

ML: manuscript writing. YB-S, DA, NM, and DG: manuscript revision. All authors have read and approved the final version of the manuscript.
